# Stable Zinc Electrode/Separator Interface Enabled by Phthalocyanine‐Modified Separator for Advanced Zinc Metal Batteries

**DOI:** 10.1002/smll.202503907

**Published:** 2025-06-01

**Authors:** Tian Wang, Ya Xiao, Weiwei Xiang, Shaocong Tang, Jae Su Yu

**Affiliations:** ^1^ Department of Electronics and Information Convergence Engineering Institute for Wearable Convergence Electronics Kyung Hee University Yongin‐si Gyeonggi‐do 17104 Republic of Korea

**Keywords:** electrostatic field layer, phthalocyanine, separator/electrode interface, Zn metal anode

## Abstract

Aqueous zinc (Zn) metal batteries (ZMBs) have received extensive attention as potential energy storage systems due to their inherent safety and low cost. However, the practical applications of ZMBs have been hindered by parasitic side reactions and Zn dendrite formation, undermining the efficiency of Zn anodes. With continuous research on the interfacial chemistry in ZMBs, increasing research interest is begun to focus on achieving dendrite‐free Zn electrodeposition by constructing a stable separator/electrode interface. In this report, a phthalocyanine (Pc)‐modified glass fiber (Pc‐GF) separator is proposed to enhance the separator/electrode interfacial chemistry and improve the stability of Zn electrode. This functionalized Pc‐GF separator can coordinate with Zn during plating and construct an electrostatic field layer to balance the interfacial electric field and Zn ion flux, facilitating stable dendrite‐free Zn electrodeposition. Consequently, the Zn symmetric cell using a Pc‐GF separator can perform 700 h stable plating/stripping at 2.0 mA cm^−2^/2.0 mAh cm^−2^. Moreover, the Zn//I_2_ full cell using a Pc‐GF separator exhibites a capacity retention of 89.0% after 500 cycles at 0.5 C and a stable operation at 5.0 C for 6000 cycles. This approach provides an effective strategy to achieve a stable Zn electrode/separator interface in aqueous ZMBs.

## Graphical abstract

This work proposes a phthalocyanine‐modified glass fiber separator to enhance the separator/electrode interfacial chemistry and improve the stability of the Zn anode. Phthalocyanine in the functionalized separator coordinates with Zn during plating, forming an electrostatic field layer on the Zn anode surface to balance the interfacial electric field and Zn ion flux, which enables planar and dendrite‐free Zn electrodeposition.

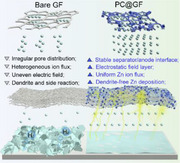

## 1 Introduction

Rechargeable aqueous zinc (Zn) metal batteries (ZMBs) have garnered widespread attention because of their intrinsic safety, low cost, and the merits of metallic Zn anode with impressive theoretical capacity (5855 mAh L^−1^, 820 mAh g^−1^) and low redox potential (‐0.76 V vs. standard hydrogen electrode).^[^ ^1^ ^]^ Nevertheless, the unstable electrode/electrolyte interface results in sluggish transfer kinetics and rapid dendrite growth which severely affect the Zn plating/stripping Coulombic efficiency and the long‐term stability of batteries, thus hindering the practical application.^[^ ^2^ ^]^ Notably, the uncontrollable dendrite growth will further damage the electrode/electrolyte interfacial chemistry and even penetrate the separator, leading to battery failure.^[^ ^3^ ^]^ To overcome these challenges, various strategies have been proposed to address unfavorable reactions on the Zn anode, focusing on the construction of artificial interface layers,^[^ ^4^ ^]^ electrode structure optimization,^[^ ^5^ ^]^ and electrolyte engineering,^[^ ^6^ ^]^ which emphasizes manipulated electrode/electrolyte interfacial chemistry to redistribute the interfacial electric field and Zn ion flux, achieving long‐term reversible Zn electrochemistry.

As a key component of the battery structure, the separator plays a vital role in regulating ion transfer behavior between the cathode and anode,^[^ ^1^, ^7^ ^]^ indicating the importance of the separator/electrode interface in achieving stable Zn electrochemistry. However, in sharp contrast to intensive studies on Zn anode and electrolyte optimization, less attention has been paid to achieving excellent separator/electrode interfacial chemistry through separator modification.^[^ ^1^, ^8^ ^]^ Fortunately, with the in‐depth study of interface chemistry, more and more work has focused on constructing a stable separator/electrode interface to achieve dendrite‐free Zn electrodeposition.^[^ ^9^ ^]^ Although glass fiber (GF) is widely used as a separator in ZMBs due to its porous structure and hydrophilic nature, its disordered fiber arrangement results in an irregular pore distribution, inducing uneven Zn deposition and potentially accelerating dendrite formation.^[^ ^3^, ^7^, ^10^ ^]^ Therefore, the functional design of the GF separator has been proposed as an effective strategy to improve the separator/electrode interfacial chemistry and enhance the electrochemical performance of the ZMBs. Phthalocyanine (Pc) is a macrocyclic conjugated organic molecule with internal holes that readily coordinate with various metals.^[^ ^11^ ^]^ The resulting metal‐Pc complex possesses a large 18π‐electron conjugated system with high carrier mobility, which has been shown to enable uniform metal electrodeposition by tuning the interfacial chemistry via electrolyte additives and artificial interfacial layer strategies.^[^ ^12^ ^]^ It has been confirmed that the π‐conjugated system with abundant two‐dimensional (2D) channels can significantly increase the diffusion coefficient of Zn ions and effectively accelerate the intramolecular electron transfer rate.^[^ ^13^ ^]^ Meanwhile, it has also been reported in Zn anode interface modification, such as poly (phenazine‐alt‐pyromellitic anhydride)^[^ ^14^ ^]^ and poly(pyrene‐co‐bromated‐benzene)^[^ ^15^ ^]^ with a linear π‐conjugated structure, which could effectively prevent direct contact between water molecules and Zn electrodes, regulate the interface solvation structure, and enhance rapid Zn ion transfer kinetics. Furthermore, the protection mechanism of cobalt tetraaminophthalocyanine as an electrolyte additive for Zn electrodes has also been studied. The results showed that the planar large conjugated ring structure was preferentially adsorbed on the Zn metal anode, promoting the desolvation of hydrated Zn ions, accelerating ion diffusion kinetics, preventing side reactions, and playing a role in balancing the interfacial electric field, which thus achieves high‐rate and dendrite‐free Zn deposition.^[^ ^12b^ ^]^ These characteristics and their positive achievements in metal batteries motivate the exploration of their potential applications in separator modification.

In this work, we proposed a Pc‐modified GF (Pc‐GF) separator strategy to stabilize Zn electrode/separator interfacial chemistry. This functionalized separator was prepared via a facile vacuum filtration method, where Pc molecules coordinated with Zn during Zn deposition to form a conjugated ring structure of zinc phthalocyanine (ZnPc), thereby constructing an electrostatic field layer to balance the interfacial electric field and enable uniform Zn electrodeposition. Meanwhile, the established ZnPc electrostatic field layer effectively prevented the direct contact between the Zn electrode and electrolyte, improving the anticorrosion of the electrode and inhibiting water‐induced parasitic reactions. Benefiting from these advantages, the Pc‐GF separator with the electrostatic field layer showed improvement in symmetric and full cells. As a result, the Pc‐GF separator enabled the Zn symmetric cell to maintain stable operation. Meanwhile, the assembled Zn//Pc‐GF//I_2_ cell delivered an excellent cycle performance with a high capacity retention, indicating the potential of Pc‐modified GF separators in practical applications.

## 2 Results and Discussion

**Figure 1:**
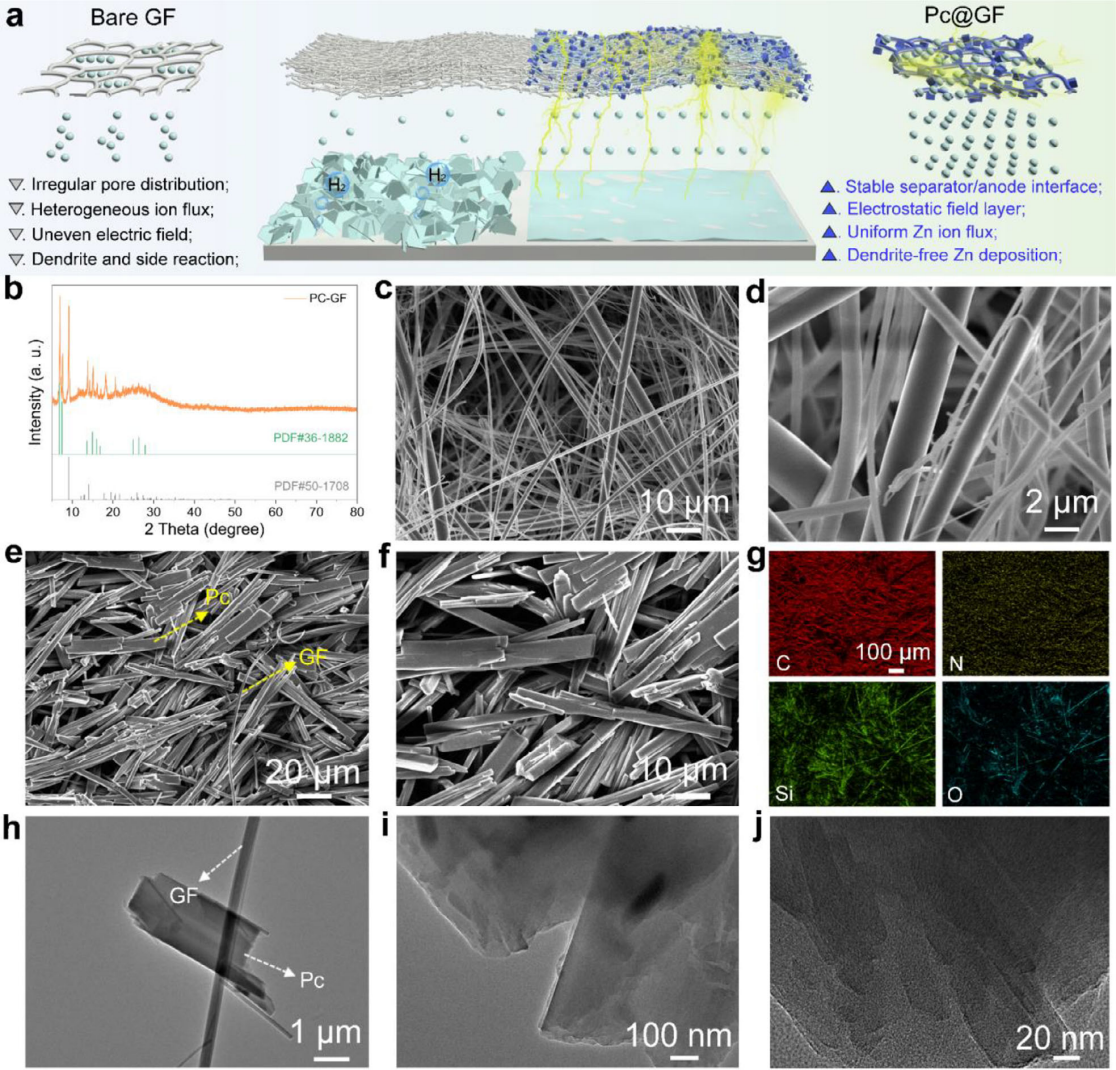
a) Schematic illustration of the Zn deposition assisted by different separators. b) XRD pattern of the Pc‐GF separator. FE‐SEM images of the c,d) bare GF, e,f) Pc‐GF separators, and g) EDS mappings of the Pc‐GF separator. h–j) HRTEM images of the Pc‐GF separator.

The effect of the Pc‐GF separator in the Zn deposition process is summarized schematically in **Figure** **1**a. For the bare GF separator, the irregular pore induces uneven Zn ion flux distribution during the Zn plating, driving inhomogeneous initial Zn nucleation. Due to the uneven electric field distribution on the electrode surface caused by the tip effect, Zn ions are further deposited at the protrusion position and promote dendrite formation. Additionally, the direct contact between the Zn electrode and the electrolyte leads to water‐induced parasitic side reactions, seriously affecting the cycling stability of the Zn electrode. In contrast, the Pc‐GF separator forms an electrostatic field layer during initial Zn deposition,^[^ ^12b^ ^]^ redistributing the interface electric field and Zn ion flux. Besides, the stabilized Zn anode/separator interface enhances electrode anticorrosion, ultimately enabling dendrite‐free Zn electrodeposition. As shown in Figure 1b, except for the characteristic peaks of the SiO_2_ (PDF#50‐1708), diffraction peaks for Pc (C_32_H_18_N_8_, PDF#36‐1882) were detected from the X‐ray diffraction (XRD) pattern of the Pc‐GF separator. Meanwhile, Figure S1 (Supporting Information) depicted the Raman spectra of the bare GF and Pc‐GF separators. Compared with the bare GF, no obvious diffraction peaks were detected. The characteristic peaks at 678, 719, 792, 1179, 1422, and 1524 cm^−1^ correspond to the benzene deformation, C─H out‐of‐plane bending, C─N─C in‐plane bending, C─H in‐plane bending, C─C stretching, and C sp^2^ vibration,^[^ ^16^ ^]^ respectively, further confirming the modification of GF by Pc molecules. The morphologies of the bare GF and Pc‐GF separators were characterized by field‐emission scanning electron microscope (FE‐SEM) analysis. Compared with the disordered fiber arrangement and irregular pore distribution of the bare GF separator (Figure 1c,d), the modified separator surface was evenly covered with Pc and retained a uniform small pores distribution architecture (Figure 1e,f), which was beneficial for coordinating the uniform distribution of Zn ion flux during Zn plating/stripping. The energy‐dispersive X‐ray spectroscopy (EDS) mappings of the bare GF and Pc‐GF separators showed clear signals of different elements, confirming that Pc was uniformly anchored on GF (Figure 1g; Figure S2, Supporting Information). The microstructure of the separator was investigated by using a high‐resolution transmission electron microscope (HRTEM). As shown in Figure 1h, bulk Pc and fibrous GF could be identified, and the corresponding magnified images could displayed the layered structure (Figure 1i,j), which is attributed to the stacking of Pc molecules.

**Figure 2:**
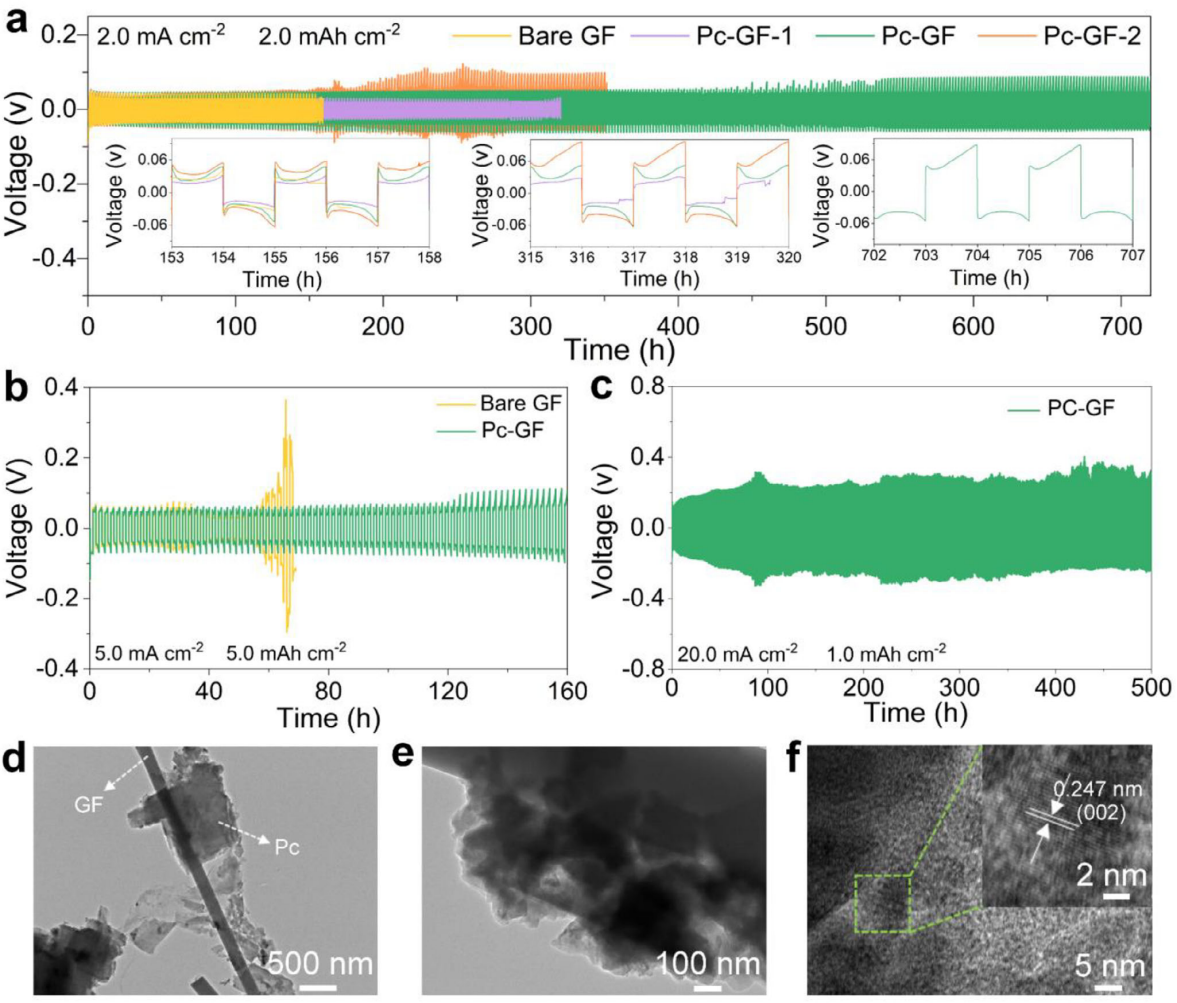
Cycling performances of the symmetric cells using different separators at a) 2.0 mA cm^−2^/2.0 mAh cm^−2^, b) 5.0 mA cm^−2^/5.0 mAh cm^−2^, and c) 20.0 mA cm^−2^/1.0 mAh cm^−2^. d–f) HRTEM images of the Pc‐GF separator after Zn deposition.

To further evaluate the effectiveness of the Pc‐GF‐induced electrostatic field layer in inducing dense and uniform Zn deposition and improving the electrochemical performance of Zn electrode, the electrochemical reversibility of the Zn symmetric cells with different separators was explored through galvanostatic cycling. The Zn symmetric cell using a Pc‐modified separator exhibited more stable Zn plating/stripping ability than the bare GF separator at 2.0 mA cm^−2^/2.0 mAh cm^−2^ (**Figure** **2**a). In detail, the Zn symmetric cell using the bare GF separator failed after 150 h. In contrast, the Zn symmetric cell using the Pc‐GF‐1 separator operated for 315 h, while using the Pc‐GF‐2 separator showed polarization after ≈200 h, which may be due to the large number of Pc molecules on the separator surface, affecting the Zn ion transport kinetics. Obviously, the Pc‐GF separator endowed the Zn electrode with an excellent plating/stripping ability, which enabled the Zn electrode to operate stably for more than 700 h. Figure S3 (Supporting Information) displays the corresponding nucleation overpotential. Although the nucleation overpotential increased with the Pc concentration, the relatively large voltage implied smaller and denser Zn deposition morphology.^[^ ^17^ ^]^ When the working condition increased to 5.0 mA cm^−2^/5.0 mAh cm^−2^ (Figure 2b), the Pc‐GF separator enabled the Zn battery to perform reversible cycling for 160 h. Meanwhile, the bare GF separator caused significant polarization of the Zn electrode after ≈50 h of plating/stripping, indicating unstable Zn deposition and severe electrode corrosion. Additionally, high‐current‐density cycling tests were conducted to demonstrate the crucial role of the stable Zn electrode/separator interface induced by the Pc‐GF separator in enhancing the stability of the Zn electrode. Benefiting from the Pc‐GF separator‐induced electrostatic shielding effect, the symmetric cell delivered stable Zn plating/stripping for 220 h at 10.0 mA cm^−2^/1.0 mAh cm^−2^, much higher than that using a bare GF separator for ≈45 h (Figure S4, Supporting Information). Furthermore, the Zn symmetric cell assembled with the Pc‐GF separator also maintained stable cycling performance for 500 h even at an extraordinary large current density of 20.0 mA cm^−2^ (Figure 2c), suggesting the rapid reaction kinetics and effective reversibility enabled by the Pc‐GF separator. Moreover, Figure S5 (Supporting Information) presents the rate performance of the Zn symmetric cells with different separators. Although the Zn electrode with the Pc‐GF separator exhibited more polarization than the bare GF separator at low current densities, the difference diminished as the current density increased, especially when the current density reached 20 mA cm^−2^. The abnormal polarization changes observed with the bare GF separator suggested that the electrostatic field layer induced by the Pc‐GF separator enhanced Zn electrode tolerance at high current density conditions. The electrochemical performance of the Zn symmetric cell using the Pc‐GF separator was compared with recently reported references (Figure S6 and Table S2, Supporting Information).^[^ ^18^ ^]^ Notably, the Zn symmetric cell using the Pc‐GF separator exhibited relatively excellent electrochemical performance, especially in the functionalized separators, suggesting that this strategy is beneficial to enhance the Zn electrode/separator interface chemistry. Additionally, the structural evolution of the Pc‐GF separator after 0.5 mAh cm^−2^ Zn deposition was investigated using HRTEM analysis. As shown in Figure 2d, the bulk and fibrous structures of Pc and GF were completely retained. Interestingly, uniformly arranged nanodots were observed with a lattice spacing of 0.247 nm, corresponding to the (002) plane of Zn (Figure 2f),^[^ ^19^ ^]^ confirming that the Pc‐GF separator facilitated uniform Zn deposition.

**Figure 3:**
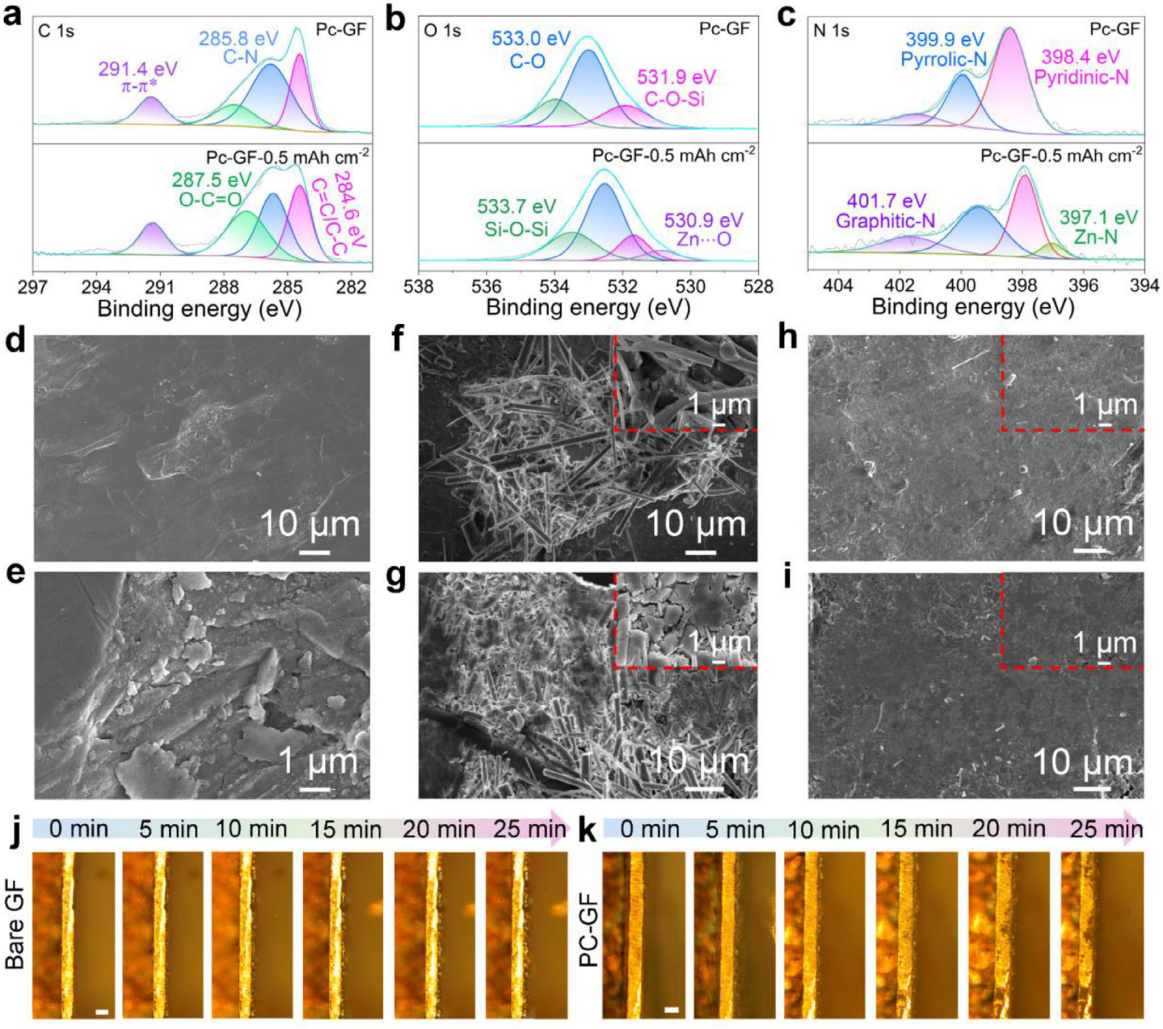
High‐resolution XPS core‐level spectra of a) C 1s, b) O 1s, and c) N 1s for the Pc‐GF separators before and after Zn plating. FE‐SEM images of d,e) the Zn electrode, the f) 1.0 mAh cm^−2^ Zn and g) 3.0 mAh cm^−2^ Zn deposited on the Zn electrode using the bare GF separator, and the h) 1.0 mAh cm^−2^ Zn and i) 3.0 mAh cm^−2^ Zn deposited on the Zn electrode using the bare Pc‐GF separator. j,k) In situ observation of the Zn deposition. (Scale bar, 100 µm).

To investigate the transformation of Pc molecules into ZnPc during Zn deposition and to construct an electrostatic field layer for balancing the interfacial electric field and achieving homogeneous Zn deposition, the surface chemicals of the Pc‐GF separator before and after Zn deposition were analyzed by X‐ray photoelectron microscopy (XPS) and the morphological evolution of the Zn electrode surface was observed using FE‐SEM images. As shown in **Figure** **3**a, the high‐resolution C 1s spectra were deconvoluted into four peaks at 284.6, 285.8, 287.5, and 291.4 eV, respectively, attributable to the C═C/C─C, C─N, O─C═O, and *π–π* ^*^ transition peaks.^[^ ^20^ ^]^ In the O 1s spectra, the Zn‐plated Pc‐GF separator was deconvoluted into four peaks at 533.7, 533.0, 531.9, and 530.9 eV, respectively, assigned to the Si─O─Si, C─O, C─O─Si, and Zn─O bonds (Figure 3b).^[^ ^9^, ^21^ ^]^ Notably, the presence of Zn─O bond in the Pc‐GF separator after Zn deposition suggested an interaction between ZnPc and water molecules, which could effectively prevent electrode corrosion and enhance the electrode electrochemical performance.^[^ ^14^ ^]^ The XPS spectra of Si 2p are displayed in Figure S7 (Supporting Information), showing no obvious change before and after Zn plating. Meanwhile, the N 1s spectrum of the Pc‐GF separator displayed three peaks at 398.4, 399.9, and 401.7 eV, corresponding to the pyridinic‐N, Pyrrolic‐N, and Graphitic‐N, respectively.^[^ ^22^ ^]^ Interestingly, another peak related to the Zn‐N bond could be detected at 397.1 eV for the Pc‐GF separator after Zn plating (Figure 3c),^[^ ^23^ ^]^ implying that the Zn coordinated with the Pc molecules to form ZnPc. The large conjugated ring architecture induced an electrostatic field layer on the Zn electrode surface, effectively improving the corrosion resistance of the Zn electrode.^[^ ^12b^ ^]^

Besides, the effect of different separators on the Zn deposition behavior was further investigated. Commercial Zn foil exhibited a rough surface structure due to defects introduced during the manufacturing process (Figure 3d,e), which aggravated the Zn electrode/separator interface instability when working with a bare GF separator, resulting in uneven electric field distribution on the electrode surface and inducing random Zn deposition.^[^ ^24^ ^]^ Figure 3f displays the surface morphology of the Zn electrode after plating 1.0 mAh cm^−2^ Zn in a symmetric cell assembled using a bare GF separator. The Zn electrode surface exhibited obvious dendrites, severely damaging the structure of the bare GF separator, which resulted in the coexistence of broken GF and dendrites. This phenomenon is mainly attributed to the Zn electrode surface defects and the uneven pore distribution of the bare GF separator, which severely disturbs the electric field distribution and Zn ion flux on the electrode surface, inducing the tip effect and subsequent dendrite formation.^[^ ^25^ ^]^ Meanwhile, as shown in Figure 3g, when the deposition capacity increased to 3.0 mAh cm^−2^, except for the dendrite problem, it could be observed from the insert that the broken GF was anchored in the newly deposited metal structure, resulting in many cracks on the electrode surface, which increased the risk of corrosion. However, the electrode obtained using the Pc‐GF separator revealed a smooth and dense deposition morphology (Figure 3h), and no damaged GF residues were found even at a deposition capacity of 3.0 mAh cm^−2^ (Figure 3i). Additionally, the electrode surface morphologies of Zn symmetric cells assembled with different separators after 20 cycles were investigated. For the Zn electrode using a bare GF separator, the remaining broken GF and moss‐like Zn dendrites on the electrode surface were observed (Figure S8a,b, Supporting Information). Meanwhile, the electrodes obtained using the Pc‐GF separator exhibited dense and uniform Zn deposition behavior, further confirming the superiority of the Pc‐GF separator (Figure S8c,d, Supporting Information). This result indicated that the employed Pc‐GF separator could form a stable Zn electrode/separator interface to regulate the uniform Zn ion flux, and the generated electrostatic field layer balanced the interfacial electric field, facilitating dense and dendrite‐free deposition. In situ optical microscopy more intuitively described the effect of the electrostatic field layer formed by the Pc‐GF separator on the Zn deposition behavior. At a current density of 5 mA cm^−2^, some small protrusions appeared on the Zn electrode surface using the pristine GF separator after performing for 10 min. Zn ions preferred to gather at the protrusions due to the heterogeneous diffusion process as the deposition time increased. The Zn deposits presented a loose and uneven deposition morphology, and the corresponding electrode thickness changed significantly, dramatically impairing the stability of the Zn electrode (Figure 3j). In contrast, the Zn electrode using the Pc‐GF separator displayed a relatively uniform Zn deposition morphology without individual protrusions during the whole Zn plating process, suggesting that the electrostatic field layer facilitated smooth and dense dendrite‐free Zn deposition (Figure 3k).

**Figure 4:**
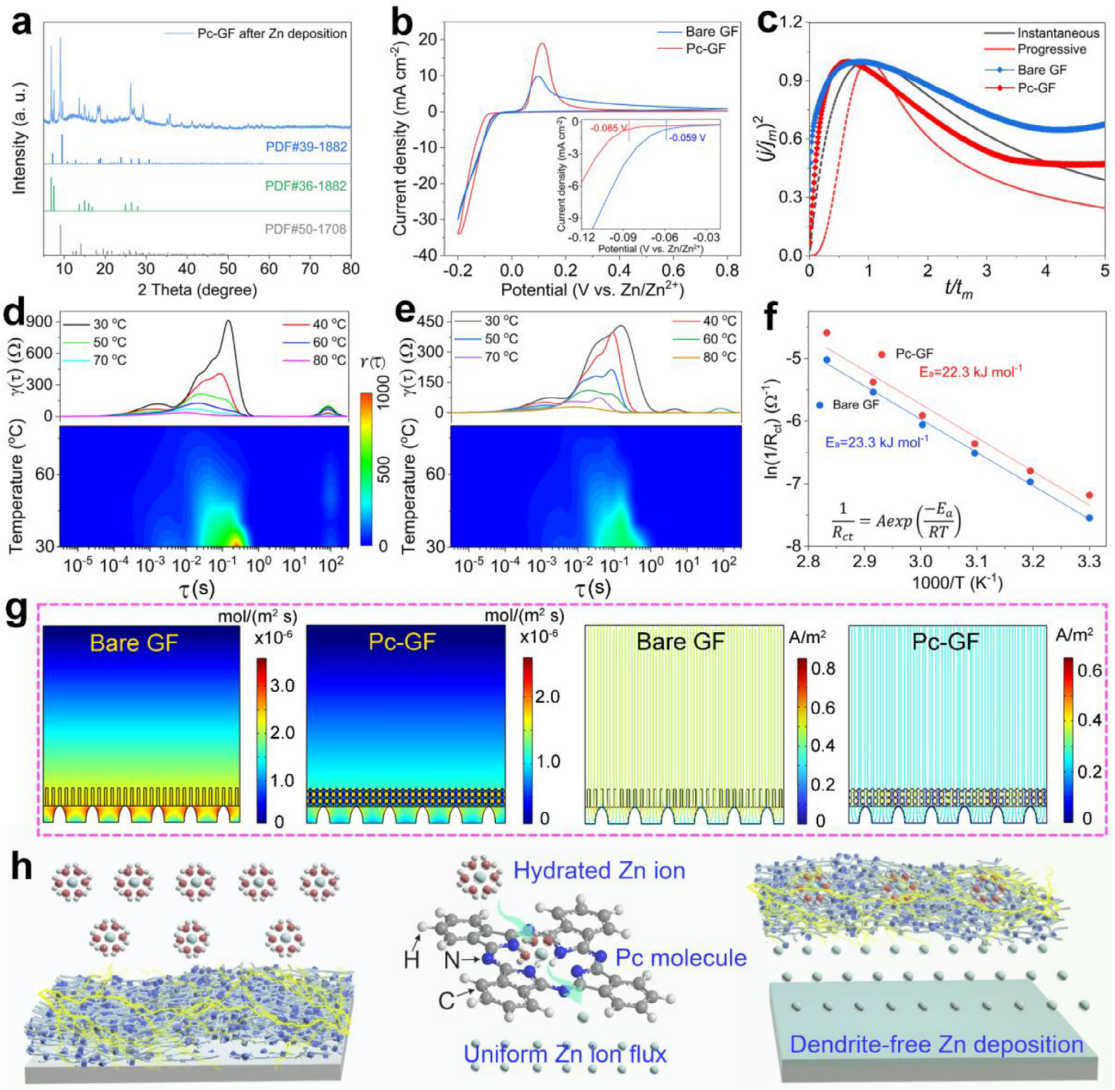
a) XRD pattern of the Pc‐GF separator after Zn deposition. b) CV curves and c) theoretical and experimental dimensionless (*j*/*j*_*m*_)^2^‐(*t*/*t*_*m*_) transients for nucleation model comparision using different separators. DRT results calculated from EIS with different temperatures and corresponding contour plots of the symmetrical Zn cells using the d) bare GF and e) Pc‐GF separators. f) Calculated activation energies. g) Simulated results of the Zn ion concentration and electric field distribution using different separators. h) Schematic diagram of the dendrite‐free Zn deposition induced by the Pc‐GF separator.

The ionic conductivity of the Ti//Pc‐GF//Ti cell was measured to be 4.2 mS cm^−1^, which is higher than that of the Ti//GF//Ti cell (3.2 mS cm^−1^), suggesting that the Pc‐GF separator can facilitate Zn ion migration (Figure S9, Supporting Information). As shown in Figure S10 (Supporting Information), the mechanical properties of the GF before and after modification were evaluated using a tensile test. The tensile strength of the Pc‐GF separator was higher than that of the bare GF separator, suggesting excellent adaptability to volume changes during Zn plating/stripping and beneficiating for resisting the penetration of Zn dendrites.^[^ ^26^ ^]^ To confirm that the Pc modification strategy improves the structural stability of the separator, Zn symmetric cells using different separators after 20 cycles were disassembled and monitored. As shown in the optical photographs in Figure S11 (Supporting Information), compared with the uneven residue of the original bare GF separator on the Zn electrode surface, Pc‐GF retained a more complete structure, suggesting its durability in regulating uniform interfacial Zn deposition. **Figure** **4**a shows the XRD pattern of the Pc‐GF separator after plating 1.0 mAh cm^−2^ Zn in a symmetric cell. In addition to the diffraction peaks of the SiO_2_ and Pc, the newly appeared peaks at 7.05, 9.4, 18.2, 18.7, and 23.7° are attributed to ZnPc (PDF#39‐1882), confirming the coordination of Pc with Zn during Zn deposition. The cyclic voltammetry (CV) curves were measured to reveal the initial Zn nucleation of Zn//Ti cells using different separators (Figure 4b). Compared to the bare GF separator, the Zn//Ti cell assembled with the Pc‐GF separator delivered a higher nucleation overpotential of 26 mV, suggesting that the electrostatic field layer formed by the Pc‐GF separator increased the Zn nucleation sites during the initial plating process.^[^ ^27^ ^]^ To reveal the Zn ion diffusion mode, a chronoamperometry (CA) test was employed on Zn symmetric cells using different separators at a constant voltage of −150 mV (Figure S12, Supporting Information). Compared with the 2D Zn ion diffusion of the bare GF separator, the current density of the symmetric cell assembled with the Pc‐GF separator was gradually stabilized after 30 s, indicating that the Pc‐GF separator facilitated a stable Zn electrode/separator interface and primarily enabled a 3D Zn ion diffusion mechanism.^[^ ^28^ ^]^ Additionally, the influence of the different separators on the Zn nucleation and growth mechanism was further investigated by the CA test at constant potential. As shown in Figure S13 (Supporting Information), the increase in current density was the enlargement of nuclei and the generation of new nuclei. The subsequent decrease represented the overlap and diffusion of adjacent nuclei.^[^ ^29^ ^]^ Besides, the CA data were further normalized to (*j/j_m_*)^2^‐(*t/t_m_*) curves and analyzed by the Scharifker‐Hills model. According to the time scale of nucleation, it was divided into instantaneous nucleation and progressive nucleation. The expressions are displayed as follows:^[^ ^30^ ^]^ ${\left(\frac{j}{j_{m}}\right)}^{2}=1.9542{\left(\frac{t}{t_{m}}\right)}^{-1}{\left\{1-exp[-1.2564(\frac{t}{t_{m}})]\right\}}^{2}$(Instantaneous nucleation), ${\left(\frac{j}{j_{m}}\right)}^{2}=1.2254{\left(\frac{t}{t_{m}}\right)}^{-1}{\left\{1-exp[-2.3367{\left(\frac{t}{t_{m}}\right)}^{2}]\right\}}^{2}$(Progressive nucleation), where *j* and *t* are the current density and time, while *j_m_* and *t_m_* represent the maximum current density and corresponding time, respectively. Therefore, the corresponding dimensionless (*j/j_m_*)^2^‐(*t/t_m_*) transient curves of using the different separators are shown in Figure 4c. The GF separator exhibited an instantaneous nucleation process, indicating that the nucleation sites were rapidly depleted in the early stages. The Zn ions tended to grow at the original nucleation sites, resulting in Zn dendrite form.^[^ ^31^ ^]^ In contrast, Zn deposition with the assistance of Pc‐GF presented both instantaneous nucleation and progressive nucleation, implying that the nucleation sites were progressively activated as the nuclei grew, which facilitated uniform and dense Zn deposition behavior.^[^ ^31^, ^32^ ^]^

Meanwhile, the reaction kinetics of the Zn symmetric cells assembled with different separators were investigated using the distribution of relaxation times (DRT) obtained by electrochemical impedance spectroscopy (EIS) analysis at a series of temperatures. The peaks located at the relaxation time (τ) of 10^−4^–10^0^ s and 10^2^ s corresponded to the charge transfer and interface diffusion resistances, respectively.^[^ ^3^, ^33^ ^]^ The Zn symmetric cell with the bare GF separator (Figure 4d) exhibited a higher charge transfer impedance than that with the Pc‐GF separator (Figure 4e), indicating that Pc‐GF facilitated charge transfer and contributed to uniform electron transfer. In addition, the interface diffusion resistance of the Zn symmetric cell assembled with the Pc‐GF separator was more sensitive to temperature changes than that of the bare GF. This further indicates that the Pc‐GF separator reduced the interface diffusion energy barrier of Zn ions and facilitated the homogeneous Zn ion diffusion. It has been confirmed that the desolvation of hydrated Zn ion was coupled with the interface charge transfer, and the energy reflected the ion deposition kinetics, which could be approximated by the activation energy (*E_a_*) calculated using the Arrhenius equation:^[^ ^34^ ^]^ $\frac{1}{R_{\mathrm{ct}}}=\mathrm{Aexp}\left(\frac{-E_{a}}{\mathrm{RT}}\right)$, where *R_ct_*, *A*, *R*, and *T* represent the interfacial resistance, frequency factor, gas constant, and absolute temperature, respectively. The EIS and the *R_ct_* of the Zn symmetric cell with different separators are shown in Figure S14 and Table S3 (Supporting Information). Correspondingly, the Zn symmetric cell assembled with the Pc‐GF separator displayed a smaller *E_a_* (22.3 kJ mol^−1^) than that using the bare GF separator (23.3 kJ mol^−1^, Figure 4f), suggesting that the Pc‐GF separator reduced Zn ion desolvation energy barrier and promoted the ion transfer kinetics at the Zn electrode/separator interface. Meanwhile, COMSOL simulation (Supporting Information) was performed to study the effect of the electrostatic field layer on the redistribution of the interface electric field and ion flux (Figure 4g). The bare GF separator had difficulty maintaining uniform Zn ion flux and electric field distribution on the electrode surface. The concentration and electric field tended to accumulate at the tip sites, resulting in uneven Zn deposition and inducing subsequent dendrite growth. Notably, the Pc‐GF separator could effectively avoid the tip effect and realize the redistribution of Zn ion flux and electric field, promoting dendrite‐free Zn metal deposition. Based on the above discussion, the Pc‐GF‐induced Zn deposition mechanism is summarized as shown in Figure 4h. Pc molecules were uniformly anchored onto the GF separator via vacuum filtration and successfully integrated into ZMBs. During the initial Zn deposition, Pc molecules coordinated with Zn to form ZnPc, whose macrocyclic conjugated ring structure induced an electrostatic field layer on the Zn electrode surface, guiding the redistribution of interfacial electric field and Zn ion flux. Additionally, the Pc‐GF separator established a stable interfacial chemistry, preventing direct contact between the Zn electrode and the electrolyte while lowering the desolvation energy of hydrated Zn ions. Consequently, the Pc‐GF separator facilitated rapid ion transport kinetics and exhibited excellent anticorrosion properties, enabling uniform and dendrite‐free Zn deposition.

**Figure 5:**
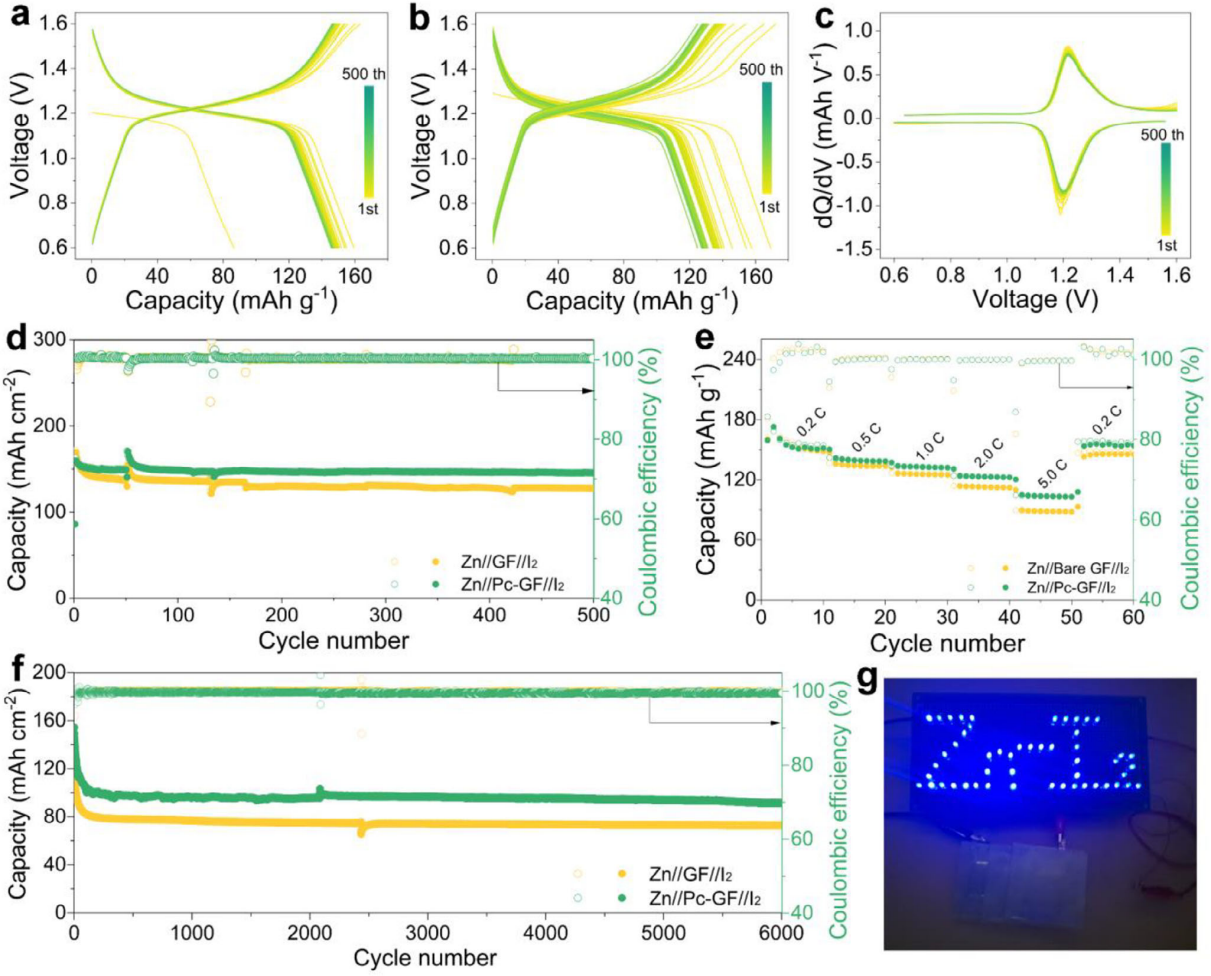
Charge/discharge curves of the a) Zn//Pc‐GF//I_2_ and b) Zn//GF//I_2_ cells. c) dQ/dV curves of the Zn//Pc‐GF//I_2_ cell. d) Cycle performance at 0.5 C, e) rate performance, and f) cycle performance at 5.0 C of the Zn//Pc‐GF//I_2_ and Zn//GF//I_2_ cells. g) Digital photograph of the Zn//Pc‐GF//I_2_ in practical application.

Therefore, Zn//I_2_ full cells were further assembled with different separators to evaluate the practicability of the Pc‐modified GF‐derived electrostatic field layer in practical applications. The charge‐discharge curves of Zn//Pc‐GF//I_2_ and Zn//GF//I_2_ cells at 0.5 C are illustrated in **Figure** **5**a,b, revealing similar initial charge capacities of 163.4 and 172.2 mAh g^−1^, respectively. The slightly lower initial charge capacity of the Zn//Pc‐GF//I_2_ cell might be attributed to the formation of an electrostatic field layer via the coordination of Pc molecules with Zn. Notably, the subsequent charge and discharge curves of the Zn//Pc‐GF//I_2_ cell showed a high overlap, whereas the Zn//GF//I_2_ battery displayed a significant capacity decrease, highlighting the positive effect of the electrostatic field layer in stabilizing Zn//Pc‐GF//I_2_ cell electrochemical performance. Figure 5c and Figure S15 (Supporting Information) show the corresponding dQ/dV curves of the two cells. Compared with the polarization of the Zn//GF//I_2_ cell, the cathodic and anodic peaks of the Zn//Pc‐GF//I_2_ remained consistent during the cycling process. This means that the electrostatic field layer on the Zn electrode surface contributed to the electrochemical reaction kinetics of Zn ions and improved the utilization of the iodine cathode.^[^ ^35^ ^]^ As shown in Figure 5d, due to the enhanced ion diffusion kinetics and anticorrosion, the Zn//Pc‐GF//I_2_ cell displayed stable cycling with a specific capacity of 145.5 mAh g^−1^ after 500 cycles and higher capacity retention of 89.0%, superior to that of the Zn//GF//I_2_ cell of 127.4 mAh g^−1^ and 73.9%, respectively. Meanwhile, the rate performance (Figure 5e) of the Zn//Pc‐GF//I_2_ cell exhibited the capacities of 154.7, 139.1, 132.9, 122.8, and 103.7 mAh g^−1^ at the current densities of 0.2, 0.5, 1.0, 2.0, and 5.0 C, respectively, higher than those of the Zn//GF//I_2_ cell (152.3, 133.8, 125.4, 112.9, and 88.7 mAh g^−1^, respectively). Additionally, the performance of the Zn//Pc‐GF//I_2_ cell was tested under a higher current density of 5.0 C (Figure 5f; Figure S16, Supporting Information). The Zn//Pc‐GF//I_2_ cell also exhibited excellent long‐term stable electrochemical performance of 91.7 mAh g^−1^ after 6000 cycles. However, the capacity of the Zn//GF//I_2_ cell only remained at 79.2 mAh g^−1^. The enhanced performance confirmed that Pc‐modified GF in the derived electrostatic field layer redistributed the electric field and Zn ion flux on the Zn anode surface and improved the Zn anode anti‐corrosion performance. Moreover, three Zn//Pc‐GF//I_2_ pouch cells connected in series could easily power more than 50 blue light‐emitting diodes (Figure 5g), suggesting their stability in practical applications.

## 3 Conclusion

In summary, we constructed a stable Zn electrode/separator interface using a Pc‐modified GF separator through a facile vacuum filtration method. During the Zn plating, the Pc molecules coordinated with Zn and formed an electrostatic field layer on the Zn electrode surface, which enabled the redistribution of the electrode surface electric field and Zn ion flux. Meanwhile, the functionalized separator could reduce the Zn ion desolvation energy barrier to promote the ion transfer kinetics and enhanced electrode anticorrosion, ultimately facilitating stable dendrite‐free Zn metal deposition. Therefore, the Pc‐GF separator enabled the Zn anode to perform stable plating/stripping for 700 h at the working condition of 2.0 mA cm^−2^/2.0 mAh cm^−2^. Meanwhile, the formed robust Zn electrode/separator interface enhanced the tolerance of the Zn electrode at high current density. Moreover, compared with the bare GF, the Zn//I_2_ full cell using the Pc‐GF separator exhibited higher capacity and long‐term cycling stability, indicating the potential of Pc‐modified GF separators in practical applications and suggesting an efficient strategy to develop long‐life ZMBs. This work provides new insight into developing stable Zn electrode/separator interfaces, paving a viable pathway for long‐term stable ZMBs.

## 4 Experimental Section

### Preparation of Pc‐GF Separator

The Pc‐GF separator was prepared via a facile vacuum filtration method. Briefly, 5.0, 10.0, and 15.0 mg Pc (98%, Sigma–Aldrich) and 2.0 mg polyvinylidene fluoride (PVDF, Sigma–Aldrich) powder were uniformly dispersed in a 10 ml N‐methylpyrrolidone (NMP, 99%, Sigma–Aldrich) solution, corresponding Pc‐GF‐1, Pc‐GF, and Pc‐GF‐2, respectively. Then, the dispersed Pc solution was slowly dripped onto the bare GF separator (Cytiva, Whatman GF/D) surface by vacuum‐assisted filtration. Subsequently, the modified GF separator was dried in a vacuum oven at 60 °C to obtain the Pc‐GF separator.

### Preparation of I_2_ Cathode

The I_2_ was anchored in activated carbon (AC) by the I_2_ sublimation method. I_2_ and AC were mixed in a mass ratio of 1:1 and heated under a sealed condition at 80 °C to prepare I_2_‐loaded AC. Subsequently, the obtained I_2_‐loaded AC was mixed with PVDF in a mass ratio of 9:1, and NMP was added to prepare a uniform slurry, which was coated on Ti foil and dried in a vacuum oven at 40 °C to obtain an I_2_ cathode. The electrode was punched into circle disks with a diameter of 12 mm, and the I_2_ loading for each disk was ≈1.0–1.3 mg.

### Material Characterizations

Based on different separators, different capacities were plated on the Zn electrode, and then the 2032‐type coin cell was disassembled. Various material analyses were performed after cleaning and drying the above‐obtained Zn electrodes or separators. XRD was carried out by using a D8 Advance (Bruker) instrument. Raman spectra were recorded by inVia Raman microscopes with an excitation wavelength of 514 nm. The morphologies of the samples were characterized by using a FE‐SEM (MERLIN (Carl Zeiss)). The surface chemicals of the different elements were analyzed by XPS (K‐alpha).

### Electrochemical Measurements

Symmetric cells were assembled using different separators, 2 M ZnSO_4_ as the electrolyte, and Zn metal foil. CV, CA, and EIS measurements were conducted on an Ivium Stat electrochemical workstation. The CV of Zn//Ti cells with different separators were studied at a scan rate of 5.0 mV s^−1^. Galvanostatic charge/discharge and dQ/dV curves of the cells were measured using a WonaTech Automatic cell test instrument (WBCS3000). EIS data were recorded over a frequency range of 0.01 to 100 kHz. Zn/I_2_ cells were cycled at 0.6 and 1.6 V and all the measurements were performed at room temperature.

## Conflict of Interest

The authors declare no conflict of interest.

## Supporting information



Supporting Information

## Data Availability

The data that support the findings of this study are available from the corresponding author upon reasonable request.
