# Supplementary material for: Stable Zinc Electrode/Separator Interface Enabled by Phthalocyanine‐Modified Separator for Advanced Zinc Metal Batteries
Source: Small. 2025 Jun 1;21(31):2503907. doi: 10.1002/smll.202503907 (PMC12332818; doi:10.1002/smll.202503907)
Supplement: Supplementary file 1 — Supporting Information [file SMLL-21-2503907-s001.docx]

Supporting Information

**Stable Zinc Electrode/Separator Interface Enabled by Phthalocyanine-Modified Separator for Advanced Zinc Metal Batteries**

Tian Wang, Ya Xiao, Weiwei Xiang, Shaocong Tang, Jae Su Yu*

Department of Electronics and Information Convergence Engineering, Institute for Wearable Convergence Electronics, Kyung Hee University, Yongin-si, Gyeonggi-do 17104, Republic of Korea

Corresponding Author: jsyu@khu.ac.kr (J. S. Yu)

**Computational Simulations**

The ion concentration and electric field simulations were conducted using the tertiary current density distribution module of the COMSOL Multiphysics. The model dimensions were set to 60 μm × 60 μm, with the vertical edges defined as insulating boundaries. Vertically arranged squares in the model represented the bare glass fiber (GF), while the phthalocyanine (Pc)-modified GF presented an orderly-arranged polygonal structure. The initial concentration of the electrolyte was 2 M, the overpotential between the substrate and electrolyte was 450 mV, and the corresponding ionic conductivity was 5 S m^-1^.^[^[^1^](#_ENREF_1)^]^ It is worth noting that this model was established on ideal conditions and could not fully reflect the real system. The current density was calculated using the Butler-Volmer equation:^[^[^2^](#_ENREF_2)^]^

$$i_{loc}=i_{0}\left( exp\left( \frac{\alpha_{a}F\eta}{RT} \right)-exp\left( \frac{{-\alpha}_{c}F\eta}{RT} \right) \right)$$

where *i_loc_*, *i_0_*, *α_a_*, and *α_c_* are the local and exchange current densities, respectively and *α_a_*, *α_c_*, and *η* are the transfer coefficients of the electrodes and the activation overpotential, respectively. The *R* and *T* represent the ideal gas constant and the temperature of 298 K, respectively.

The relation between the diffusion coefficient and electric mobility follows the Nernst-Einstein equation:^[^[^2-3^](#_ENREF_2)^]^

$$N_{i}=-D_{i}{\nabla c}_{i}-z_{i}u_{m,i}{Fc}_{i}{\nabla\phi}_{l}+{uc}_{i}$$

where *Z_i_* is the Zn ion transfer number and *u_m,i_* is the electric mobility coefficient. F and *ϕ* represent the Faraday constant and potential, respectively. *N_i_* and *D_i_* represent the Zn ion flux and the diffusion coefficient, respectively.

Table S1. Parameters setting for the COMSOL simulation.

| **Name** | **Value** | **Description** |
| --- | --- | --- |
| *σ_1_* | 5.0 S m^-1^ | Ionic conductivity (Electrolyte) |
| *σ_2_* | 4.2 mS cm^-1^ | Ionic conductivity (Pc-GF) |
| *T_0_* | 298 K | System temperature |
| *C_0_* | 2 M | Initial electrolyte concentration |
| *i0_ref_* | 2.0 A m^-2^ | Exchange current density |
| *Z* | 2 | Charge number |
| *P_Zn_* | 7.14 g cm^-3^ | Density |
| *M* | 65.0 g mol^-1^ | Molar mass |
| *D* | 1.5 × 10^-8^ m^2^ s^-1^ | Diffusion coefficient |
| *Phil* | 0 V | Initial electrolyte potential |
| *Ф* | 0.1 V | Balance potential |
| *Ф* | 450 mV | Overpotential |
| *C_lim_* | 1.0 × 10^-3^ mol m^-3^ | Concentration limit |
| *R* | 8.314 J·mol·K^-1^ | Molar gas constant |


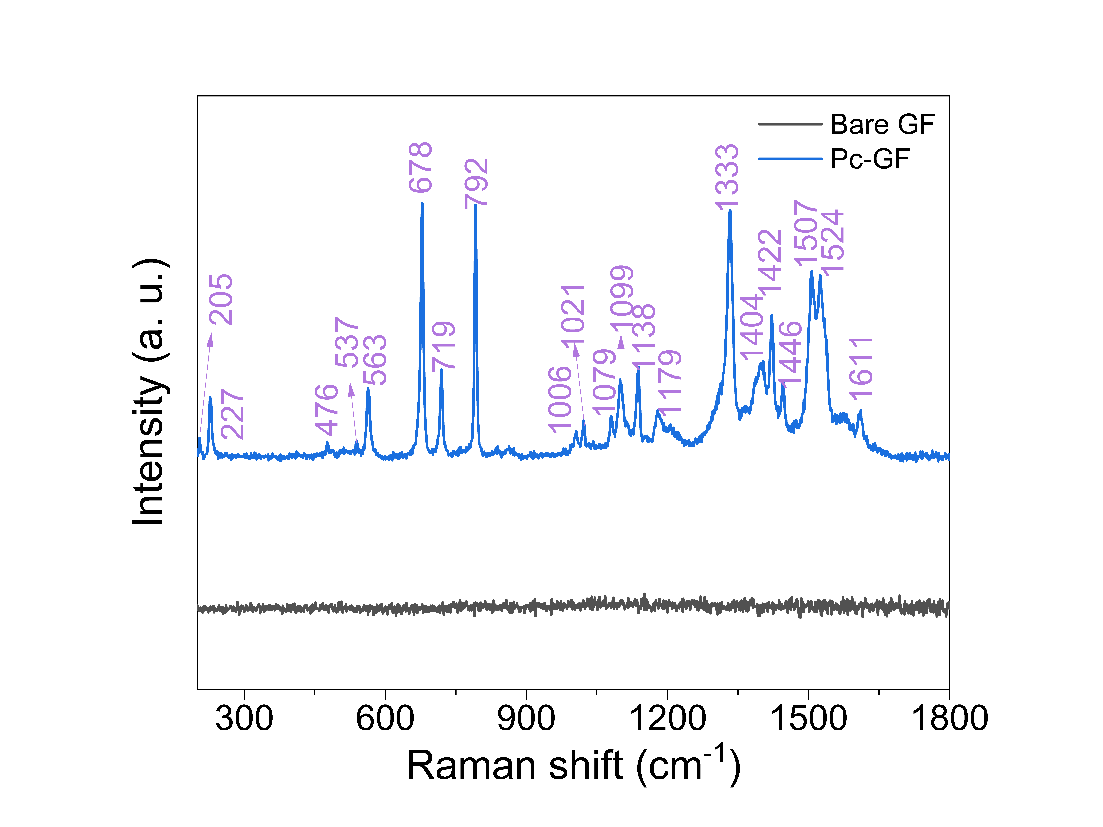


Figure S1. Raman spectra of the bare GF and Pc-GF separators.


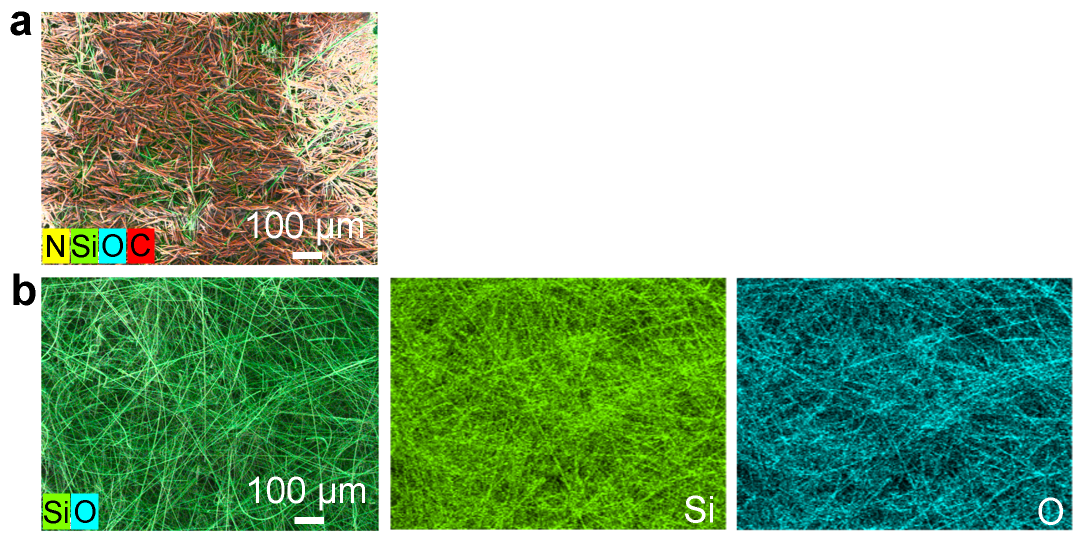


Figure S2. EDS mappings of the (a) Pc-GF and (b) bare GF separators.


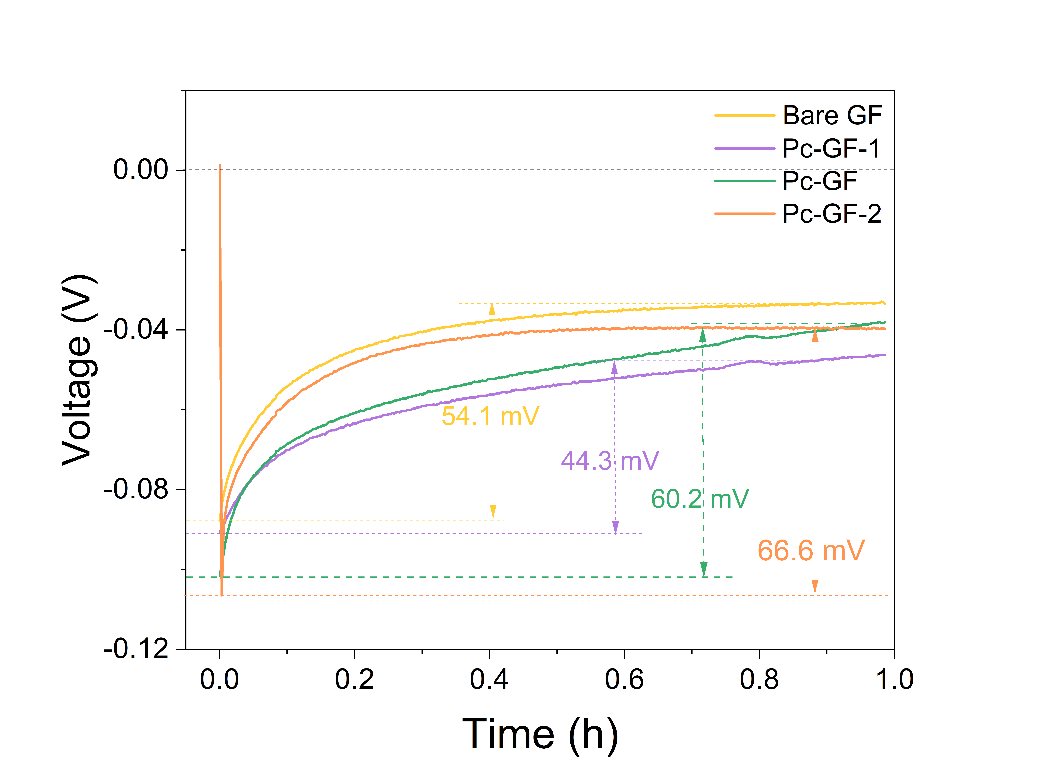


Figure S3. Voltage-time curves of Zn deposition using the bare GF, Pc-GF-1, Pc-GF, and Pc-GF-2 at 2.0 mA cm^-2^.


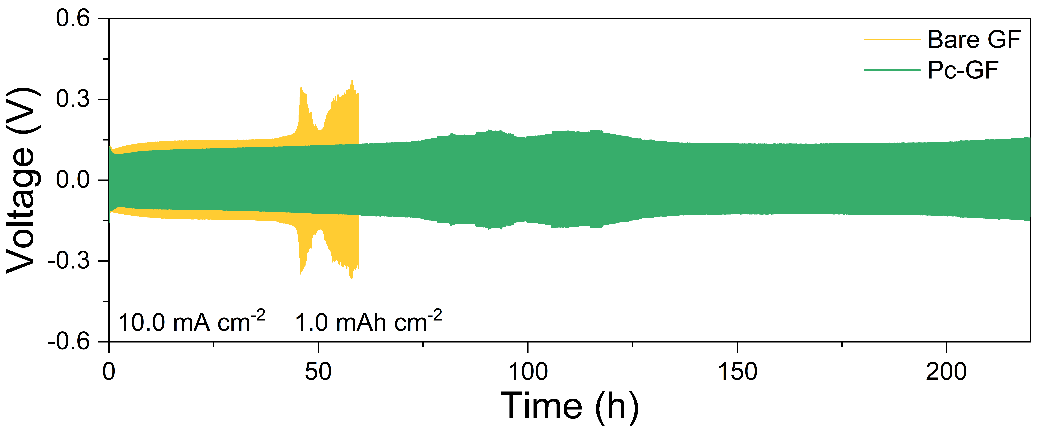


Figure S4. Cycling performance of the symmetric cells using the bare GF and Pc-GF separator.


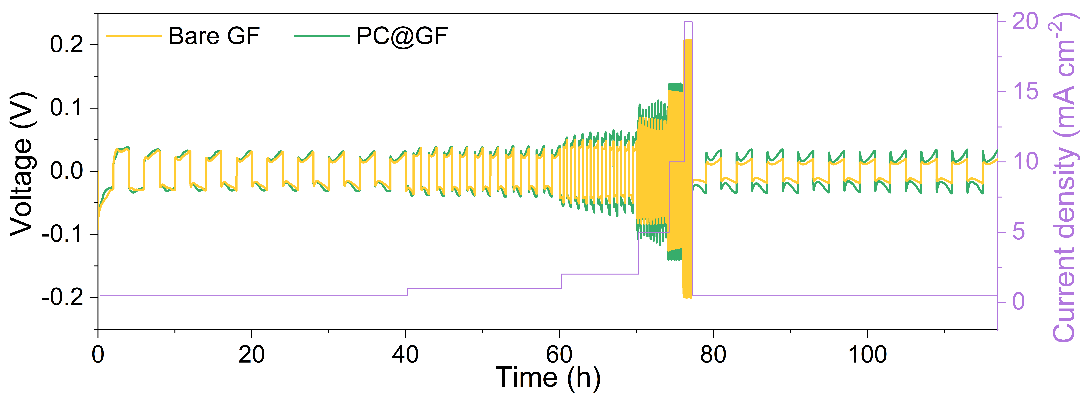


Figure S5. Rate performance of the symmetric cells using different separators.


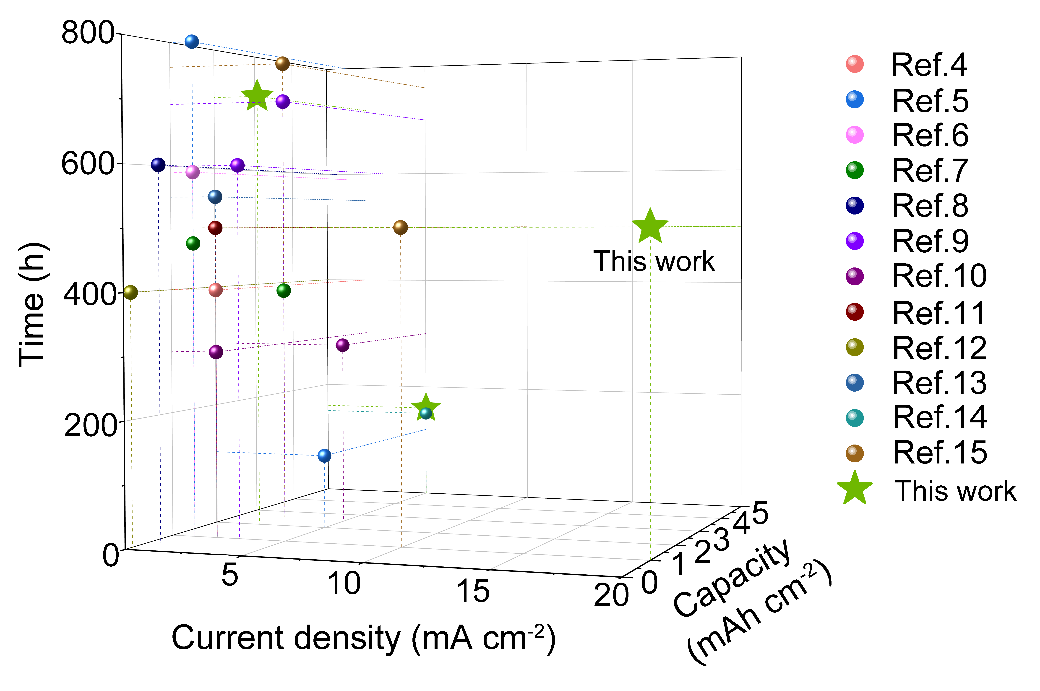


Figure S6. Comparative electrochemical performance of the Zn symmetric cell using the Pc-GF separator with other reported literatures.

Table S2. Comparative electrochemical performance of the Zn symmetric cell using the Pc-GF separator with the previously reported literature.

|  | **Method** | **Current density (mA cm^-2^)** | **Capacity (mA cm^-2^)** | **Time (h)** | **Ref.** |
| --- | --- | --- | --- | --- | --- |
| 3D In@Zn@In | Electroless plating coupled with electrodepositing | 2.0 | 1.0 | 400 | ^[^[^4^](#_ENREF_4)^]^ |
| P-CN | Surface coating | 1.0 | 1.0 | 800 | ^[^[^5^](#_ENREF_5)^]^ |
|  |  | 5.0 | 2.0 | 120 |  |
| Threonine | Electrolyte additive | 1.0 | 1.0 | 590 | ^[^[^6^](#_ENREF_6)^]^ |
|  |  | 5.0 | 1.0 | 700 |  |
| Poly(ether ether ketone) | Spin coating | 1.0 | 1.0 | 475 | ^[^[^7^](#_ENREF_7)^]^ |
|  |  | 5.0 | 1.0 | 400 |  |
| Cyclized PAN | Surface coating | 0.5 | 0.5 | 600 | ^[^[^8^](#_ENREF_8)^]^ |
| CaF_2_ | Surface coating | 3.0 | 1.0 | 600 | ^[^[^9^](#_ENREF_9)^]^ |
|  |  | 5.0 | 1.0 | 700 |  |
| Na_3_V_2_(PO_4_)_3_ | Surface coating | 2.0 | 1.0 | 300 | ^[^[^10^](#_ENREF_10)^]^ |
|  |  | 5.0 | 2.5 | 300 |  |
| Kaolin | Functionalized separator | 2.0 | 1.0 | 500 | ^[^[^11^](#_ENREF_11)^]^ |
| C_3_N_4_ | Functionalized separator | 0.1 | 0.1 | 400 | ^[^[^12^](#_ENREF_12)^]^ |
| TiO_2_ | Fiber membrane | 2.0 | 1.0 | 550 | ^[^[^13^](#_ENREF_13)^]^ |
| Ferroelectric polymer | Membrane | 5.0 | 5.0 | ~150 | ^[^[^14^](#_ENREF_14)^]^ |
| Filter paper | Wet strength promotion | 5.0 | 1.0 | 760 | ^[^[^15^](#_ENREF_15)^]^ |
|  |  | 10.0 | 1.0 | ~500 |  |
| Pc-GF | Functionalized separator | 2.0 | 2.0 | 720 | This work |
|  |  | 5.0 | 5.0 | 160 |  |
|  |  | 20.0 | 1.0 | 500 |  |


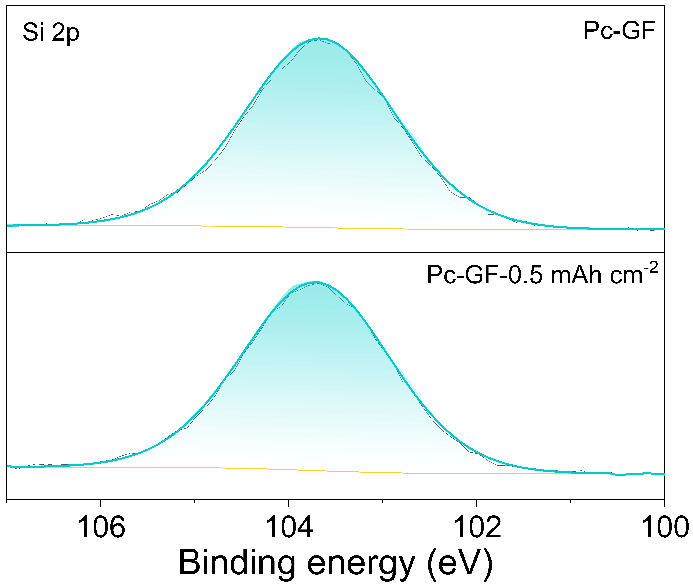


Figure S7. XPS spectra of the Si 2p of Pc-GF separator before and after Zn plating.


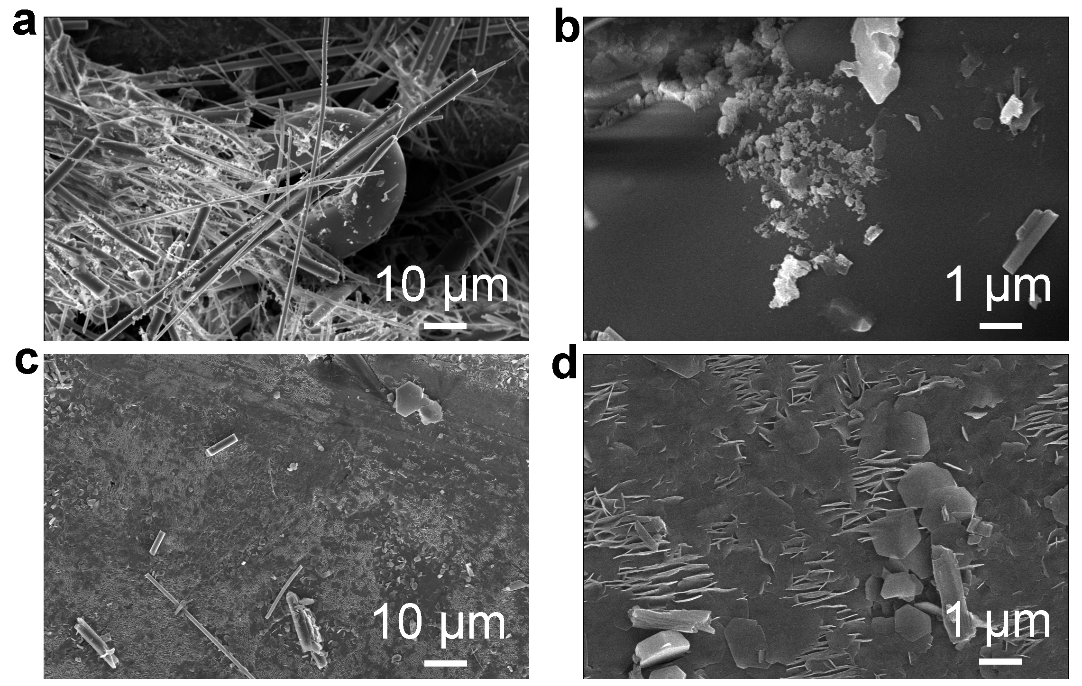


Figure S8. FE-SEM images of Zn electrode after 20 cycles using the (a, b) bare GF and (c, d) Pc-GF separators.


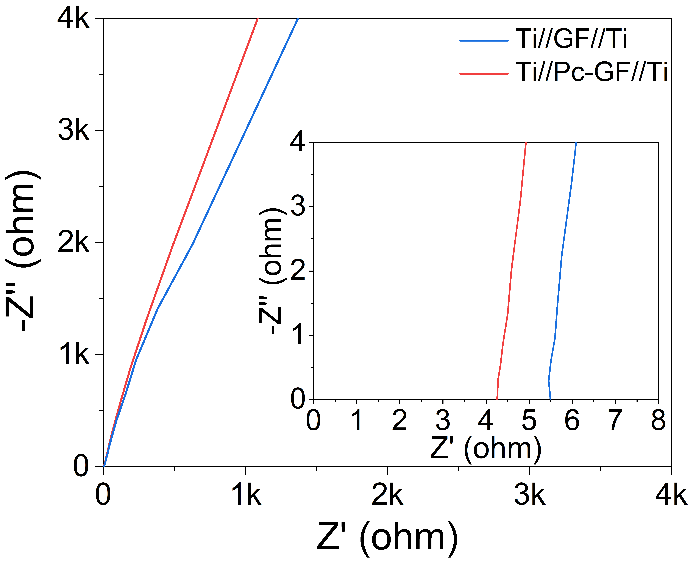


Figure S9. Nyquist plots of the Ti//GF//Ti and Ti//Pc-GF//Ti cells (inset: enlargement of the indicated curves).

The ionic conductivity (σ) was calculated by the following equation:

$$\sigma=\frac{L}{R_{b}S}$$

where *L*, *R_b_*, and *S* represent the thickness of the separator, the resistance of Pc-GF, and the effective contacting area.^[^[^16^](#_ENREF_16)^]^


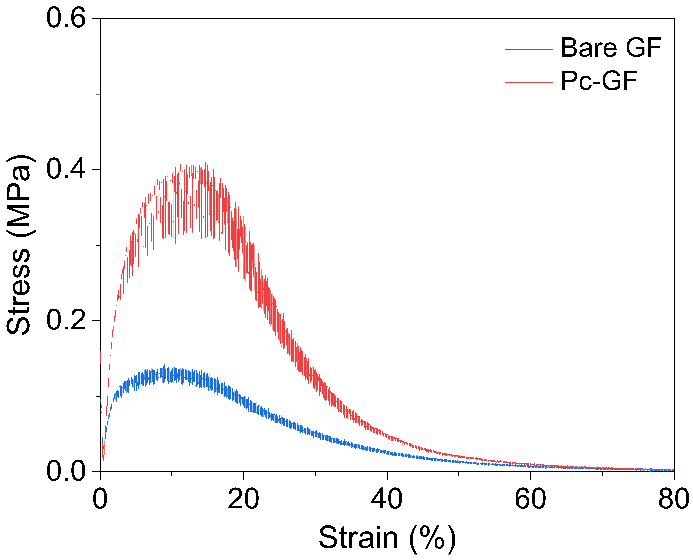


Figure S10. Tensile stress-strain curves of the bare GF and Pc-GF separators.


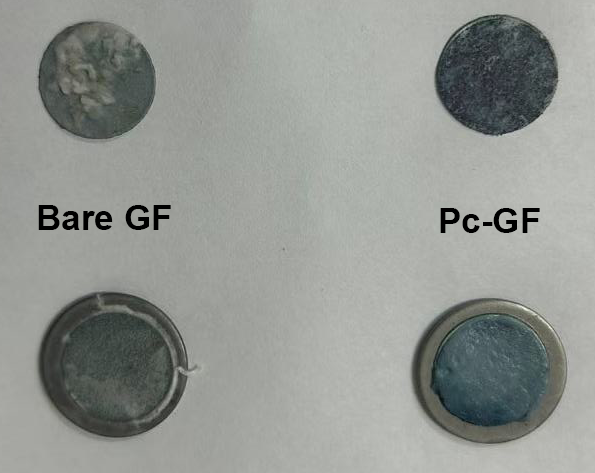


Figure S11. Optical photographs of the Zn symmetric cells using different separators after 20 cycles.


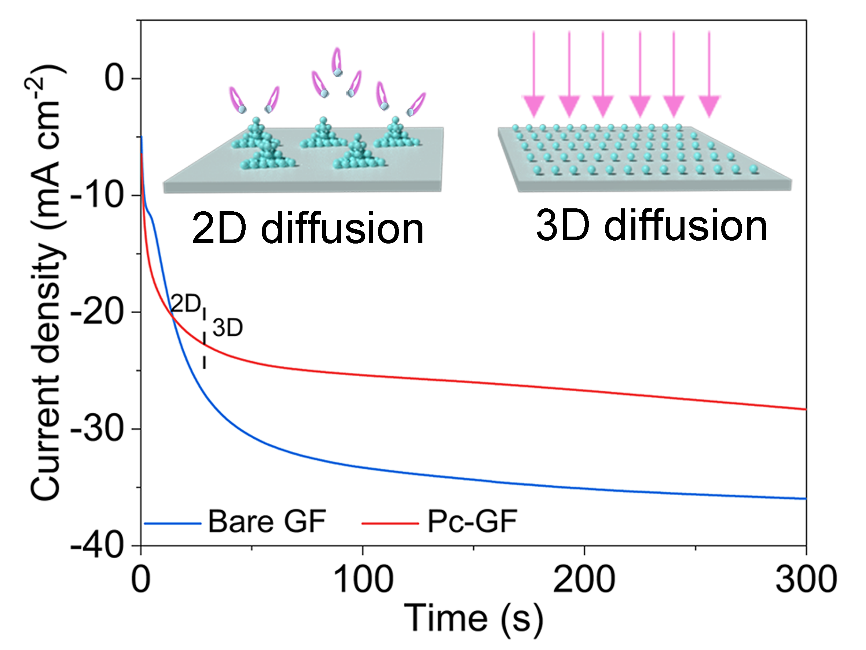


Figure S12. CA curves using different separators at a constant voltage of -150 mV.


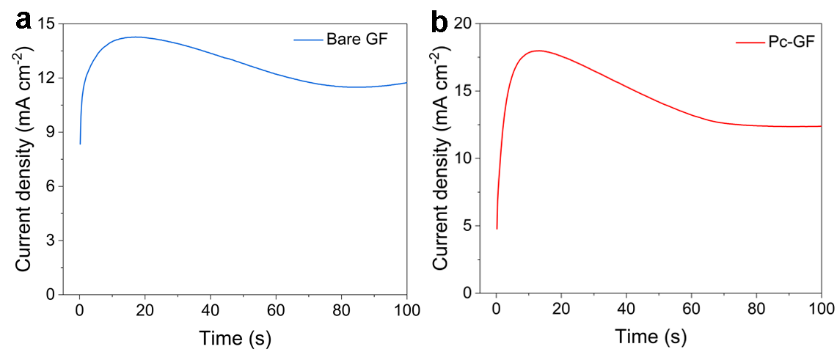


Figure S13. Current-time transients obtained at predetermined potentials for the (a) bare GF and Pc-GF separators.


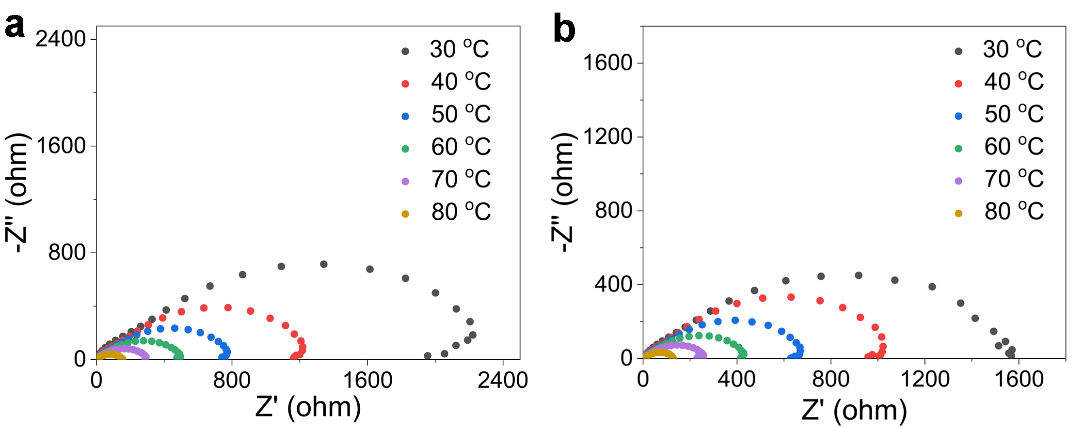


Figure S14. EIS curves of the Zn systematic cells using the (a) bare GF and (b) Pc-GF separators at different temperatures.

Table S3. Fitted R*_ct_* values of the Zn systematic cells using (a) bare GF and (b) Pc-GF separators at different temperatures.

| **T (K)** | **R*_ct_* (ohm)** | **ln (R*_ct_*^-1^) (ohm^-1^)** |
| --- | --- | --- |
| Bare GF |  |  |
| 303 | 1892 | -7.54 |
| 313 | 1063 | -6.97 |
| 323 | 671.7 | -6.51 |
| 333 | 427.7 | -6.06 |
| 343 | 253.6 | -5.54 |
| 353 | 151.4 | -5.02 |
| Pc-GF |  |  |
| 303 | 1317 | -7.18 |
| 313 | 891.2 | -6.79 |
| 323 | 578.9 | -6.36 |
| 333 | 368.1 | -5.91 |
| 343 | 216 | -5.37 |
| 353 | 98.4 | -4.59 |


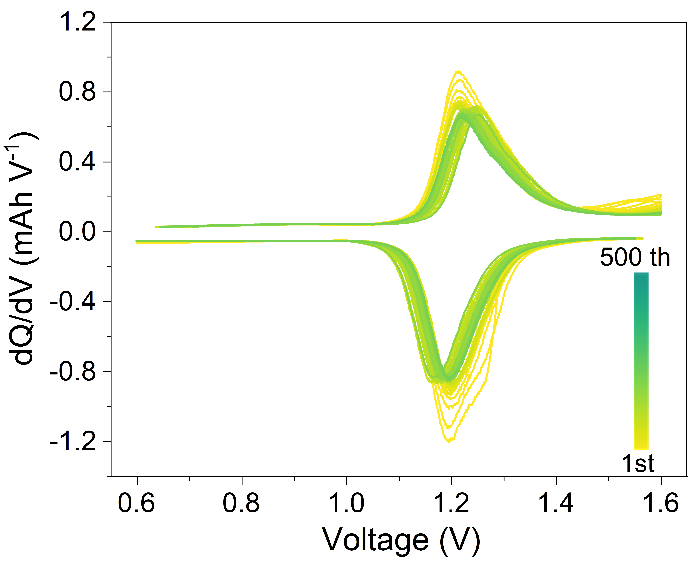


Figure S15. dQ/dV curves of the Zn//GF//I_2_ cell.


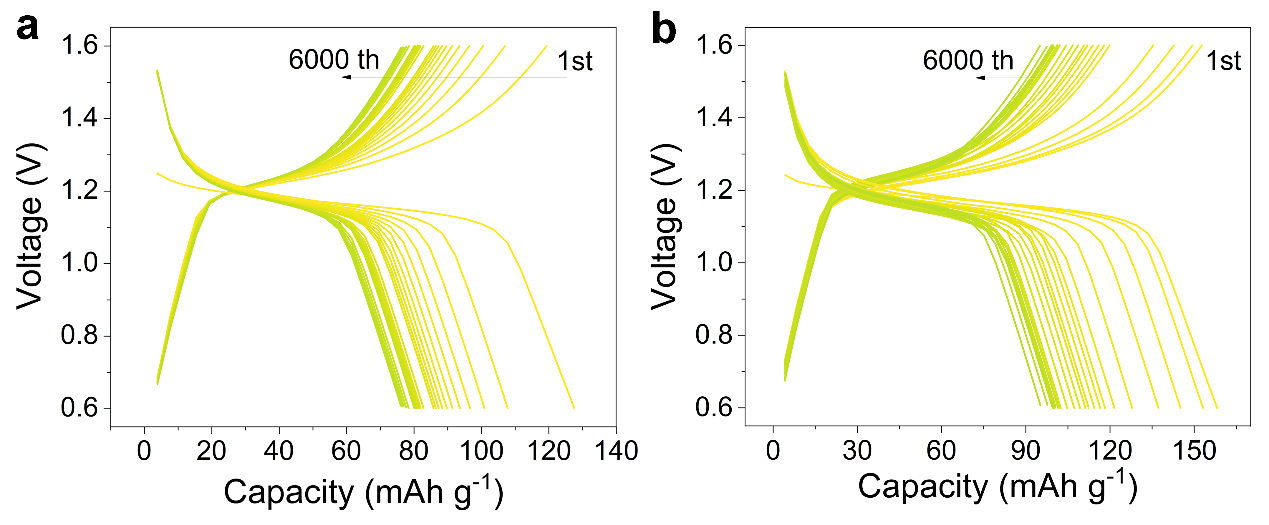


Figure S16. Charge/discharge curves of the (a) Zn//GF//I_2_ and (b) Zn//Pc-GF//I_2_ cells.

**References**

[1] S. Zhai, N. Wang, X. Tan, K. Jiang, Z. Quan, Y. Li, Z. Li, *Adv. Funct. Mater.* **2021**, *31*, 2008894.

[2] Q. Cao, Y. Gao, J. Pu, X. Zhao, Y. Wang, J. Chen, C. Guan, *Nat. Commun.* **2023**, *14*, 641.

[3] Q. Cao, Z. Pan, Y. Gao, J. Pu, G. Fu, G. Cheng, C. Guan, *Adv. Funct. Mater*. 2022, *32*, 2205771.

[4] X.-Y. Fan, H. Yang, B. Feng, Y. Zhu, Y. Wu, R. Sun, L. Gou, J. Xie, D.-L. Li, Y.-L. Ding, *Chem. Eng. J*. **2022**, *445*, 136799.

[5] Y. Xiao, T. Su, T. Wang, W. Xiang, S. Tang, J. S. Yu, Chem. Eng. J. **2025**, *512*, 162391.

[6] Z. Miao, Q. Liu, W. Wei, X. Zhao, M. Du, H. Li, F. Zhang, M. Hao, Z. Cui, Y. Sang, X. Wang, H. Liu, S. Wang, *Nano Energy* **2022**, *97*, 107145.

[7] Q. Jian, Y. Wan, Y. Lin, M. Ni, M. Wu, T. Zhao, ACS Appl. Mater. Interfaces **2021**, *13*, 52659.

[8] J. Yang, S. Wang, L. Du, S. Bi, J. Zhu, L. Liu, Z. Niu, *Adv. Funct. Mater.* **2024**, *34*, 202314426.

[9] Y. Feng, Y. Wang, L. Sun, K. Zhang, J. Liang, M. Zhu, Z. Tie, Z. Jin, *Small* **2023**, *19*, 2302650.

[10] N. Guo, Z. Peng, W. Huo, Y. Li, S. Liu, L. Kang, X. Wu, L. Dai, L. Wang, S. C. Jun, Z. He, *Small* **2023**, *19*, 2303963.

[11] N. Zhao, Y. Liang, W. Huo, X. Zhu, Z. He, Z. Zhang, Y. Zhang, X. Wu, L. Dai, J. Zhu, L. Wang, Q. Zhang, *Chinese Chem. Lett.* **2024**, *35*, 109332.

[12] R. Sun, P. Xia, X. Guo, S. Dong, F. Xu, Y. Zhang, S. Lu, Q. Zheng, H. Fan, Chem. Eng. J. **2024**, *486*, 150377.

[13] Y. Liu, X. Zhang, J. Rong, D. Li, S. Lv, X. Sun, *J. Energy Storage* **2025**, *114*, 115886.

[14] Y. Dong, W. Liu, C. Carlos, Z. Zhang, J. Li, F. Pan, J. Sui, X. Wang, *Nano Lett*. **2024**, *24*, 4785.

[15] L. Li, M. Sun, B. Hao, W. Chen, C. Zhu, L. Zhang, X. Shen, X. Zhou, J. Zhou, C. Yan, X. Liu, T. Qian, *J. Phys. Chem. Lett.* **2024**, *15*, 380.

[16] a) P. Zou, R. Zhang, L. Yao, J. Qin, K. Kisslinger, H. Zhuang, H. L. Xin, *Adv. Energy Mater.* **2021**, *11*, 2100982; b) T. Wang, L. Xu, W. Xiang, S. Tang, Y. Xiao, J. S. Yu, *Adv. Energy Mater.* **2024**, *14*, 2402586.
